# Clotting state after cardioversion of atrial fibrillation: *a haemostasis index could detect the relationship with the arrhythmia duration*

**DOI:** 10.1186/1477-9560-3-2

**Published:** 2005-03-06

**Authors:** Eleni Hatzinikolaou-Kotsakou, Zafirios Kartasis, Dimitrios Tziakas, Dimitrios Stakos, Athanasios Hotidis, Georgios Chalikias, Georgios Bourikas, Dimitrios I Hatseras

**Affiliations:** 1 Academic Cardiology Department, Academic Hospital Dragana Alexandroupolis, Demokritus University of Thrace, Greece; 2 Academic Hematology Department, Academic Hospital Dragana Alexandroupolis, Demokritus University of Thrace-Greece

**Keywords:** Atrial fibrillation, haemostatic markers

## Abstract

**Background:**

Fibrin D-dimer levels have been advocated as an useful clinical marker of thrombogenesis.

**Hypothesis:**

We hypothesized that i) there is a hyperclotting state after the return of atrial fibrillation to sinus rhythm, ii) the measurement of plasma D-Dimer levels might be a good screening tool of this clotting status, and iii) the duration of arrhythmia influences the haemostasis measured by plasma D-Dimer levels.

**Methods:**

Forty-two patients with atrial fibrillation undergoing cardioversion were divided into two groups: in Group A (n = 24,14 male, 56 ± 11 years) the duration of atrial fibrillation was 72 hours or more (142.7 ± 103.8 hours), in Group B (n = 18, 10 male, 61 ± 13 years) the duration of atrial fibrillation was less than 72 hours (25 ± 16 hours). Plasma fibrin D-dimer levels were measured by enzyme immunoassay before, and 36 hours after, cardioversion. The change of plasma D-dimer levels 36 hours after cardioversion was calculated as delta-D-dimer.

**Results:**

There were no significant differences in demographic, clinical, and echocardiographic data, and the success of cardioversion between the two groups. Compared to the control, the baseline D-dimer levels were significantly higher in both groups. The delta D-dimer levels were significantly higher in Group A than in Group B (p < 0.005). Furthermore, plasma D-dimer levels 36 hours after cardioversion (r = 0.52, p = 0.0016) and delta-D-dimer levels (r = 0.73, p < 0.0001) showed significant correlations with the duration of atrial fibrillation.

**Conclusion:**

The longer duration of the atrial fibrillation episode could lead to a more prominent cardiovascular hyperclotting state after cardioversion, and the mean changes of plasma D-Dimer levels could be used as an useful clinical marker of the clotting state after atrial systole return.

## Introduction

Atrial fibrillation is the most common sustained arrhythmia in clinical practice. It is associated with an increased risk of thrombus formation, resulting in substantial morbidity, with the augmented risk of stroke being the most serious. This could be explained by haemostasis conditions favouring thrombosis: previous studies have demonstrated that in most patients, AF is a high risk factor for hypercoagulability, irrespective of underlying structural heart diseases or aetiology [1-8].

In addition, it is well known that the direct current cardioversion of atrial fibrillation, especially if persisting >48 h, carries a great risk of thromboembolism, which extends to 5% of cases not receiving anticoagulant therapy. The mechanism and pathogenesis of thromboembolic episodes after restoration of sinus rhythm in these patients is not completely understood.

There is some evidence that the prothrombotic state associated with atrial fibrillation might contribute towards the risk of thromboembolism following cardioversion, but reports are not clear. In this context, having a marker of coagulation activation would be useful in identifying patients at highest thromboembolic risk. Indicators of hypercoagulability, such as D-dimers, which are indicative of a prothrombotic state, might also be indicative of thromboembolic risk [9]. D-dimers, which originate from the formation and lysis of cross-linked fibrin, are therefore specific markers of coagulation activation. In AF elevated D-dimer levels have been reported to be associated with left atrial appendage dysfunction [10], and the potential presence of atrial thrombi. Anticoagulation does reduce D-dimer levels, but there were no significant correlations of D-dimer levels with either warfarin dose or the INR [11,12]. D-dimer levels may also increase as a result of comorbidity conditions causing intravascular (i.e. in thrombosis) or extravascular cross-linked fibrin turnover, such as in renal failure, liver impairment, acute or chronic infection, neoplastic disease, hypertension, acute cardiovascular syndromes, bleeding, haematoma and surgery. The interpretation of D-dimer levels can, therefore, be considered as reflecting the prothrombogenic state of patients without these acute clinical conditions, and without overt thrombosis. [9] However, the age of the patient must be considered: D-dimer levels are reported to increase with age [13-15], which makes the interpretation of D-dimer measurement very difficult and hazardous in older people. Thus, before an evaluation of the predictive role of D-dimer levels for thromboembolic events in AF patients can be made, two conditions must be fulfilled: D-dimer levels should be characteristic of each patient with AF, and the presence of co-morbidity should be excluded.

We therefore hypothesized that i) there is still a hyperclotting state after return of sinus rhythm, ii) the measurement of plasma D-dimer levels pre- and post-cardioversion might be a good screening tool of this clotting status, and iii) the duration of arrhythmia could be a good predictor of thromboembolic events after cardioversion, due to the influence on haemostasis measured by plasma D-dimer levels.

## Methods

Over a period of 18 months, we studied 42 consecutive patients, aged between 39–68 years old, with non-valvular atrial fibrillation who underwent successful electrical cardioversion and who remained in sinus rhythm at the one-month visit. Exclusion criteria were other acute causes of atrial fibrillation (for example, thyrotoxicosis, pneumonia or other infections), acute cardiovascular or cerebro-vascular events (myocardial infarction, congestive heart failure, stroke, etc) occurring within five months, valvular heart disease, malignancy, connective tissue disease, infectious or inflammatory conditions and chronic renal/hepatic disease. The patients were divided into two groups. In Group A (24 pts, 14 male, 56 ± 11 years), the duration of atrial fibrillation was 72 hours or more (142.7 ± 103.8 hours). In Group B (18 pts, 10 male, 61 ± 13 years) the arrhythmia had a duration of less than 72 hours (25 ± 16 hours). We included only patients treated with anticoagulant-coumadin for chronic prophylaxis. There was not a statistically significant difference between the mean duration of anticoagulation treatment between the two groups (14 ± 4 months for group A and 12 ± 6 months for Group B). Prothrombin time to an INR (international normalized ratio) of 2.5–3 was considered as a necessary inclusion criterion for all patients. The total population had a history of drug refractory atrial fibrillation, with a serious number of arrhythmia episodes. We only analyzed the documented arrhythmia episodes. The total number of documented episodes of paroxysmal atrial fibrillation in Group A was 33, and in Group B the patients had 36 confirmed arrhythmia episodes. There were no significant differences in age, sex, hematocrit, hemoglobin, plasma fibrinogen level, underlying heart disease, success ratio of electrical cardioversion, echocardiographic data, presence of diabetes mellitus or hypertension between the two groups. The clinical characteristics of the patients are shown in Table 1. AF was seen at the 12 lead surface electrocardiogram. We compared the D-dimer levels of these two groups at baseline, before cardioversion with a matched control group (n = 19) without atrial fibrillation. Plasma Fibrin D-dimer levels were measured before, and 36 hours after, cardioversion. Anticoagulation reduces D-dimer levels, but as we analyzed the D-dimer levels of the same patients pre- and post-cardioversion, we did not need to use a cut-off value, and all patιents were under coumadin treatment with an INR (international normalized ratio) of 2.5 to 3 at least 3 weeks before cardioversion, and 36 hours post-cardioversion. The study protocol was approved by the local ethics committee (Academic Hospital of Alexandroupolis decision 09/11/2002). All patients received oral and written information concerning the background of the study, and signed informed consent.

**Table 1:** Clinical Characteristics for Total Study Group

	**Group A (n = 24)**	**Group B (n = 18)**	**Control Group (n = 19)**
Age (years)	56 ± 11	61 ± 13	59 ± 12
Male gender	14(58%)	10(56%)	10(57%)
Smokers	15(62.5%)	11(62%)	11(62.5%)
Systolic Blood Pressure (mmHg)	145 ± 20	143 ± 22	139 ± 25
Diastolic Blood Pressure (mmHg)	80 ± 10	82 ± 11	80 ± 11
Known hypertension (160/90 mmHg)	8 (33.4%)	6 (34%)	7(35%)
Lone AF	11(45.8%)	8 (44.2%)	-
Coronary artery Disease	5(20.8%)	4(22%)	5(22.8%)
Diabetes mellitus	6(25%)	4(22%)	4(22.3%)
Hct(%)	45.8 ± 3.9	44.9 ± 4.2	45 ± 3.6
Hb(g/dl)	15.3 ± 1.34	14.8 ± 2.9	15.2 ± 3.1
Fbg (mg/dl)	229.9 ± 27.0	237 ± 37.6	227 ± 26
**P value**: non significant (NS)			
Echocardiographic data for Both Groups pre cardioversion			

		**Group A**	**Group B**

Left atrial diameter(cm)		4.2 ± 0.6	4.0 ± 0.8
Left ventricular diastolic dimension (cm)		5.2 ± 1.0	5.0 ± 0.9
Left ventricular systolic dimension (cm)		4.1 ± 0.5	4.0 ± 0.8

**P value**: non significant (NS)

### Laboratory

An intravenous line was placed and blood samples for D-dimer measurement were taken from the patients immediately before cardioversion, and 36 hours after recovery of sinus rhythm. Citrated plasma was obtained from venous blood by centrifugation at 2,500 rpm for 15 min at 4°C. Aliquots were stored at -70°C to allow batch analysis. The plasma D-dimer levels were measured by the enzyme-linked immunosorbent assay method. The measurements were obtained with the use of a quantitative sandwich immunochromatographic technique (Cardiac D-Dimer; Roche Diagnostics, Mannheim, Germany). For every blood sample, measurements were done twice. The investigators and attending physicians were blinded to the D-dimer test results. Inrta-assay coefficients of variation for assays were <5%, inter-assay variances were 10%. The changes of plasma D-dimer levels 36 hours after cardioversion were calculated as delta- D-dimer.

### Echocardiography

Echocardiographic examinations were performed in all patients, immediately prior to the procedure and 36 hours after successful cardioversion. Transthoracic echocardiographic two-dimensional imaging and guided pulsed wave Doppler recordings were obtained. Transmitral Doppler inflow velocities were recorded from the apical four-chamber view.

Peak velocities of early fillings (E) wave and atrial filling (A) wave were determined. The inter- and intra-observer variability was <5% for these measurements. We did not detect the presence of thrombi in the left atrium. However, the absence of thrombus on transthoracic echocardiography does not preclude the real absence of thrombus.

### Statistical Analysis

Clinical variables are expressed as the mean value ± SD. The effects of duration from the onset of atrial fibrillation on the measured indexes were analyzed by two-way repeated measures analysis of variance (ANOVA). Sequential data pre- and post-cardioversion were analyzed by Friedman's repeated measures analysis of variance. Correlations were performed by Spearman's rank correlation method. Stepwise multiple regression analyses were performed to determine independent predictors for plasma D-dimer levels, using age, sex, left-atrial size, left ventricular dimensions (diastole, systole) presence of underlying medical disease, smoking status, and the presence of atrial fibrillation. A p value < 0.05 was considered statistically significant. Analyses were performed with SAS for Windows 8.02 (SAS Institute Inc., Cary, North Carolina) and GraphPad Prism, version 3.00 (GraphPadSoftware, San Diego, California) statistical software packages.

## Results

The baseline D-dimer levels of each group are shown in Table 2. The D-dimer levels in the control group were significantly lower than both groups (p < 0.05) (Table 2).

**Table 2:** Mean D-Dimer levels at baseline before cardioversion in both groups and the relationship with he control group

	Group A	Group B	p value
Mean D-Dimer	117 ± 74.7 ng/ml	102 ± 53.8	NS
	Group A	Control Group	p value

Mean D-Dimer	117 ± 74.7 ng/ml	39.7 ± 28.6	0.05
	Group B	Control Group	p value
	
Mean D-Dimer	102 ± 53.8	39.7 ± 28.6	0.05

The patients' sinus rhythm was restored by applying electrical cardioversion in all study patients. Delta D-dimer levels were significantly higher in Group A than in Group B. (p < 0.005).

There were no significant differences in plasma D-dimer levels before, and 36 hours after, cardioversion between the two Groups (Table 3). Furthermore, plasma D-dimer levels 36 hours after cardioversion, and delta D-dimer levels showed significant correlations with the duration of atrial fibrillaton: D-dimer 36 hours after CV; r = 0.52, p = 0.0016, delta D-dimer; r = 0.73, p < 0.0001.

**Table 3:** D-dimer levels (ng/ml) in both Groups of Patients

	**D-dimer before CV**	**D-dimer after CV**	**delta *D-dimer***
**Group A**	*117.9 ± 74.7 ng/ml*	*104.1 ± 59.7 ng/ml*	*34.2 ± 63.6 ng/ml°*
**Group B**	***102 ± 53.8 ng/ml***	***84.7 ± 78. 2 ng/ml***	***-17.3 ± 37.8 ng/ml***

Values are expressed as *mean ± SD, °p = 0.005 vs Group B*.

The echocardiographic data 36 hours after CV have changed. There was a return of atrial contractility using Doppler echocardiography, as shown by the progressive increase in A-wave velocity (Friedman's repeated measures ANOVA, p < 0.0001).

Two embolic stroke events were recorded during the six hours after cardioversion in Group A (8,33%) and none in Group B. The embolic events occurred in two women with an INR of 2.6 and 2.9 respectively. At the onset of the event one patient had D-dimer levels of 112.2 ng/ml while the second patient had D-dimer levels of 109.8 ng/ml. Fortunately, these were transient events. The computerized tomography detected non-extentend thromboembolic areas.

## Discussion

In the present study we analyzed D-dimer levels as a screening index of a hyperclotting state after cardioversion of atrial fibrillation. It would be more useful if we used the term abnormal haemostasis, because the fibrin D-dimer is a cross-linked degradation product, resulting from the balance between thrombogenesis and the fibrinolysis process.

Fibrin D-dimer levels have been established as a useful clinical marker of thrombogenesis. [16] The use of D-Dimer levels in the investigation and management pathway of venous thromboembolism is well established. [17] This marker has a high sensitivity and specificity in excluding thromboembolism, when a well-defined assay is used in the appropriate clinical setting. [18]

In "normal" patients, elevated D-dimer levels have been associated with a higher risk of developing cardiovascular disease. [19] Also, such cross-linked fibrin degradation products have been shown to be strong and independent predictors of the severity of occlusive peripheral artery disease [20].

Patients with long-lasting episodes of atrial fibrillation or chronic atrial fibrillation are characterized by increased levels of plasma D-dimers [21,22], platelet activation, and endothelial damage and/or dysfunction, which is consistent with the increased predisposition to thrombus formation in this group of patients [23]. Some investigators have shown that the cardioversion of atrial fibrillation to sinus rhythm results in a decrease of D-dimer levels [24]. Our data showed elevated D-dimer levels in individuals who received appropriate anticoagulation therapy at the time of cardioversion. This may suggest the presence of some remaining clot. But we could not estimate this hypothesis because we did not have the profile of D-dimer levels of all patients during a long period before the time of enrolment.

In the present study, the plasma D-dimer levels decreased in both groups after successful cardioversion, but the mean change in D-dimer levels pre- and post- cardioversion was significantly lower in Group A than in Group B, and D-dimer levels continued to be high, even 36 hours after the procedure in Group A. There were reported embolic events in 8.33% of Group A patients, with the higher delta D-dimer.

These data confirm the existence of an abnormal clotting state after the cardioversion of atrial fibrillation, which could be the cause of the thromboembolic events observed after the return to sinus rhythm, even under anticoagulation.

The mechanism of thromboembolism is debatable. The results we found could suggest an embolism might be caused by detachment of a formed thrombus during the phase of passage from AF to sinus rhythm, due to being heavily compromised by stunning the mechanical function of the left atrium and left atrium appendage after sinus rhythm restoration. The stunning of the left atrium and left atrium appendage might attenuate the thrombogenetic activation which our result showed.

In the present study we also provided evidence that this hypercoaguable state after successful cardioversion of atrial fibrillation related directly to the duration of the arrhythmia episode. It has been accepted that chronic atrial fibrillation is characterized by increased levels of plasmatic D-dimers, with a wide inter-individual variability, depending on the patients and therapeutic characteristics. However, it has not been established if this level could also be characteristic of paroxysmal or persistent atrial fibrillation in patients, and whether it could be a predictive factor for the risk of thromboembolic events after cardioversion to sinus rhythm.

Our results show that longer duration of atrial fibrillation could lead to a more prominent cardiovascular hyperclotting state after cardioversion and that the duration of the arrhythmia episode might be a risk factor for the high incidence of post-cardioversion thromboembolic events. Importantly, this hypercoaguable state does not appear to be subject to any other clinical variables, nor is it related to whether or not the patient had a lone atrial fibrillation or not, suggesting that the duration of the arrhythmia episode confers a constant prothrombotic state per se, after cardioversion to sinus rhythm, which was independent of the etiology but dependent on the duration of the arrhythmia. Interestingly enough, this hyperclotting state after cardioversion exists even under appropriate anticoagulative treatment. The relation of the markers of accelerated coagulation to clinical or echocardiographic risk factors for thromboembolism is controversial.

Our results clearly demonstrate a positive correlation of the based on the arrhythmia duration clinically predicted embolic risk to the mean change of plasma D-dimer levels 36 hours after successful cardioversion. We could not detect any relation to the echocardiographic risk factors, probably due to our inability to perform a transesophageal echocardiography. The present study suggests that even in the absence of clinical conditions causing increased embolic risk, patients with signs of accelerated coagulation are at risk of thrombus formation in the future.

In conclusion, a longer duration of the atrial fibrillation episode could lead to a more prominent cardiovascular hyperclotting state after cardioversion, and the mean changes of plasma D-Dimer levels could be used as an useful clinical marker of the clotting state after atrial systole return.

### Clinical implications

Our study might have practical implications for the management of patients with an episode of atrial fibrillation, regardless of the anticoagulative treatment they are receiving: the episode must be terminated as soon as possible, because the pathogenesis of thrombus formation in atrial fibrillation is very complex and not yet completely defined. Further investigations with a large population are needed to define all the pathophysiologic mechanisms of thrombus formation.
